# Supplementary material for: A Leucyl-tRNA Synthetase Urzyme: Authenticity of tRNA Synthetase Catalytic Activities and Promiscuous Phosphorylation of Leucyl-5′AMP
Source: Int J Mol Sci. 2022 Apr 11;23(8):4229. doi: 10.3390/ijms23084229 (PMC9026127; doi:10.3390/ijms23084229)
Supplement: Supplementary file 1 [file ijms-23-04229-s001.zip › ijms-1649284-supplementary.pdf]

Gene and amino acid sequences of the two LeuAC constructs used are:

```
LeuAC_1:
GAG AAA AAA TTC TAC ATC ACC GTG GCG TTC CCG TAC ACG AGC GGC CAT CTG CAC GTT GGT CAC GCG ATT
 E   K   K   F   Y   I   T   V   A   F   P   Y   T   S   G   H   L   H   V   G   H   A   I
ACC TAT ACG ATC CCG GAT ATT ATC GCC CGT TTT AAA CGC ATG CAG GGC TAC AAT GTG CTG TTC CCG ATG
 T   Y   T   I   P   D   I   I   A   R   F   K   R   M   Q   G   Y   N   V   L   F   P   M
GCC CTG CAT ACC GAT GGT CTG ACC GAT AGT ACG ATT TAT ATG GCA GTT CTG CTG ATC CTG TAT TGG TAC
 A   L   H   T   D   G   L   T   D   S   T   I   Y   M   A   V   L   L   I   L   Y   W   Y
CCG CTG GAT TGG CGT TGC AGC GGC AAA GAT CTG ATT CCG AAC CAT CTG ACC TTT TTC ATC ATC AAC CAC
 P   L   D   W   R   C   S   G   K   D   L   I   P   N   H   L   T   F   F   I   I   N   H
GTG GCA ATC TTC CGC GAA GAA CAT TGG CCG AAA GGT ATC GCG GTT AAC GGC TTC GGT ACG CTG GAA GGC
 V   A   I   F   R   E   E   H   W   P   K   G   I   A   V   N   G   F   G   T   L   E   G
CAG AAA ATG AGC AAA TCT AAG GGT AAC GTG CTG AAT CGT ATC
 Q   K   M   S   K   S   K   G   N   V   L   N   R   I

LeuAC_2
GAA AAG AAA TTT TAT ATC ACC GTG GCC TTT CCG TAT CTG AGT GGC CAT CTG CAT GTT GGT CAT GCC CGC
 E   K   K   F   Y   I   T   V   A   F   P   Y   L   S   G   H   L   H   V   G   H   A   R
ACC TAT ACC ATT CCG GAT GAA ATT GCA CGC ACC AAA CGT AAA CAG GGC TAT AAT GTT CTG TTT CCG ATG
 T   Y   T   I   P   D   E   I   A   R   T   K   R   K   Q   G   Y   N   V   L   F   P   M
GAT TGG CAT ACC ACC AGC CTG AGC GAT AGC ACC ATC TAT ATG GCA GAA TAT ACC AGT GAA TAT TGG TAT
 D   W   H   T   T   S   L   S   D   S   T   I   Y   M   A   E   Y   T   S   E   Y   W   Y
CCG CTG GAT TGG CGC TGC AGC GGC AAA GAT CTG ATT CCG AAT CAT CTG ACC AAA TTC ATT TTT AAT CAC
 P   L   D   W   R   C   S   G   K   D   L   I   P   N   H   L   T   K   F   I   F   N   H
GTG GCA ATT TTC CGT GAA GAA CAT TGG CCG AAA GGT ATT GCC GTT AAT GGC AGT GGT ACA CTG GAA GGC
 V   A   I   F   R   E   E   H   W   P   K   G   I   A   V   N   G   S   G   T   L   E   G
CAG AAA ATG AGT AAA AGC AAA GGT AAT GTT CTG AAT TTC AGC
 Q   K   M   S   K   S   K   G   N   V   L   N   F   S
```

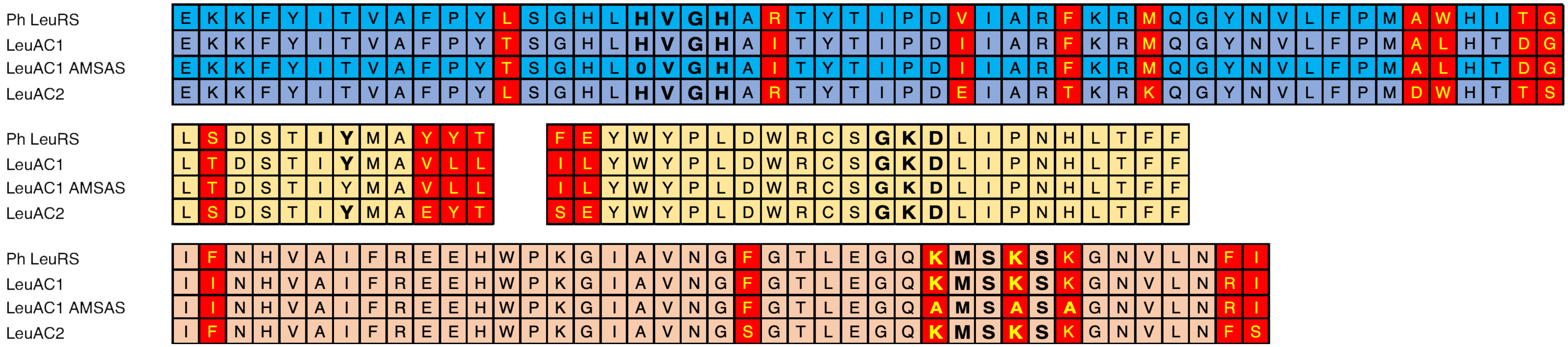

Figure S1. Sequence differences between LeuAC constructs and homologous sequence from *P. horikoshii* LeuRS. The sequence is broken into three fragments analogous to the three described by Pham, et al., (1). Connecting peptide 1 (CP1) connects the C-terminus of the blue fragment to the N-terminus of the Amber fragment. Connecting Peptide 2 (CP2) is indicated by the gap in the Amber fragment. Differences between the three sequences are highlighted in red boxes. Active site residues are highlighted in bold and larger fontsize. Entries for LeuAC2 enhance the solubility of LeuAC2, but have no detectable effect on the active site titration timecourses.

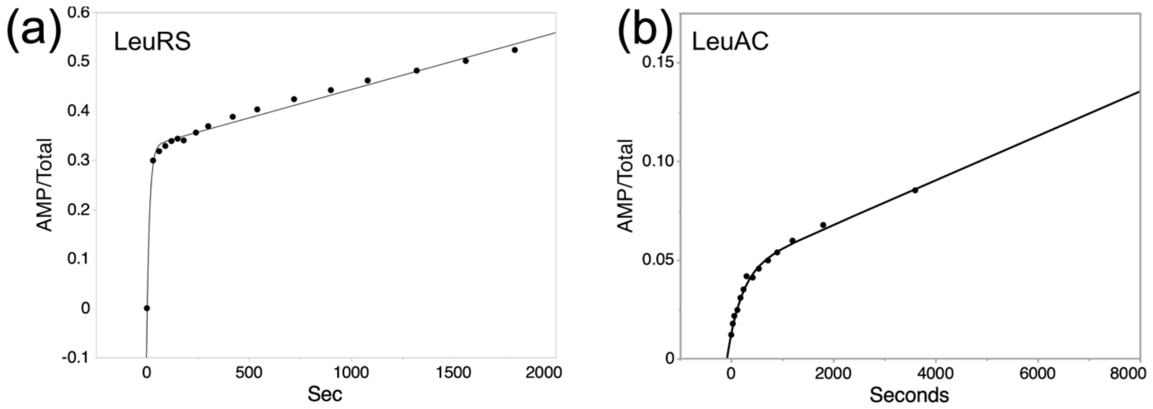

Parameter	β	σ	β/σ
C	0.32	0.00375	86.5
A	0.32	0.01003	32.3
kchem	8.1×10^{-2}	0.012	7.0
kcat	9.0×10^{-5}	4.4×10^{-6}	20.4
kchem/kcat	902		

Parameter	β	σ	β/σ
C	0.05	0.00215	21.1
A	0.03	0.0022	14.6
kchem	3.6×10^{-3}	0.00056	6.5
kcat	1.1×10^{-5}	9.5×10^{-7}	11.9
kchem/kcat	319		

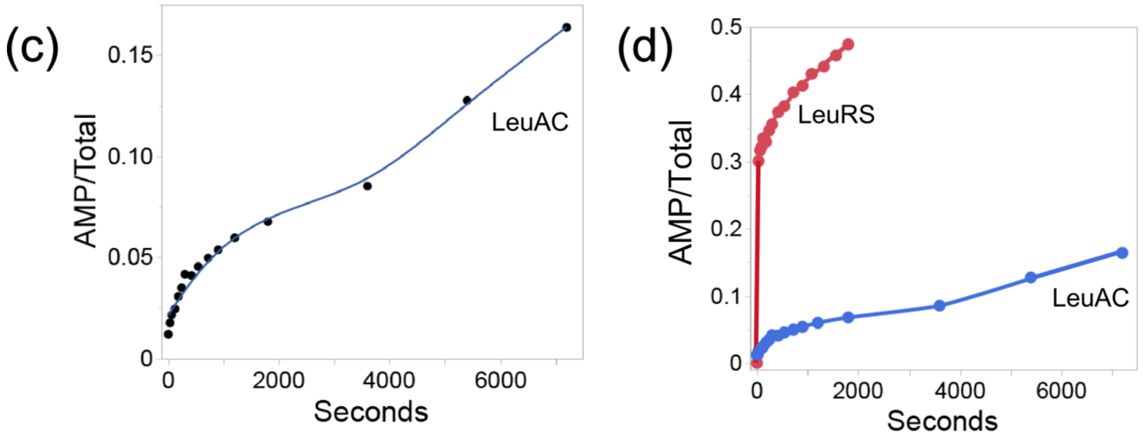

Figure S2. Timecourses for AMP formation by LeuRS and TEV-cleaved LeuAC. **(a)**. Active-site titration plot for LeuRS. **(b)**. Active-site titration plot for LeuAC, using only timepoints to 4000 seconds. **(c)**. Extended timecourse for LeuAC, showing an increased linear turnover rate from 4000 to 7200 seconds. **(d)**. LeuRS and LeuAC timecourses compared on the same coordinate system.

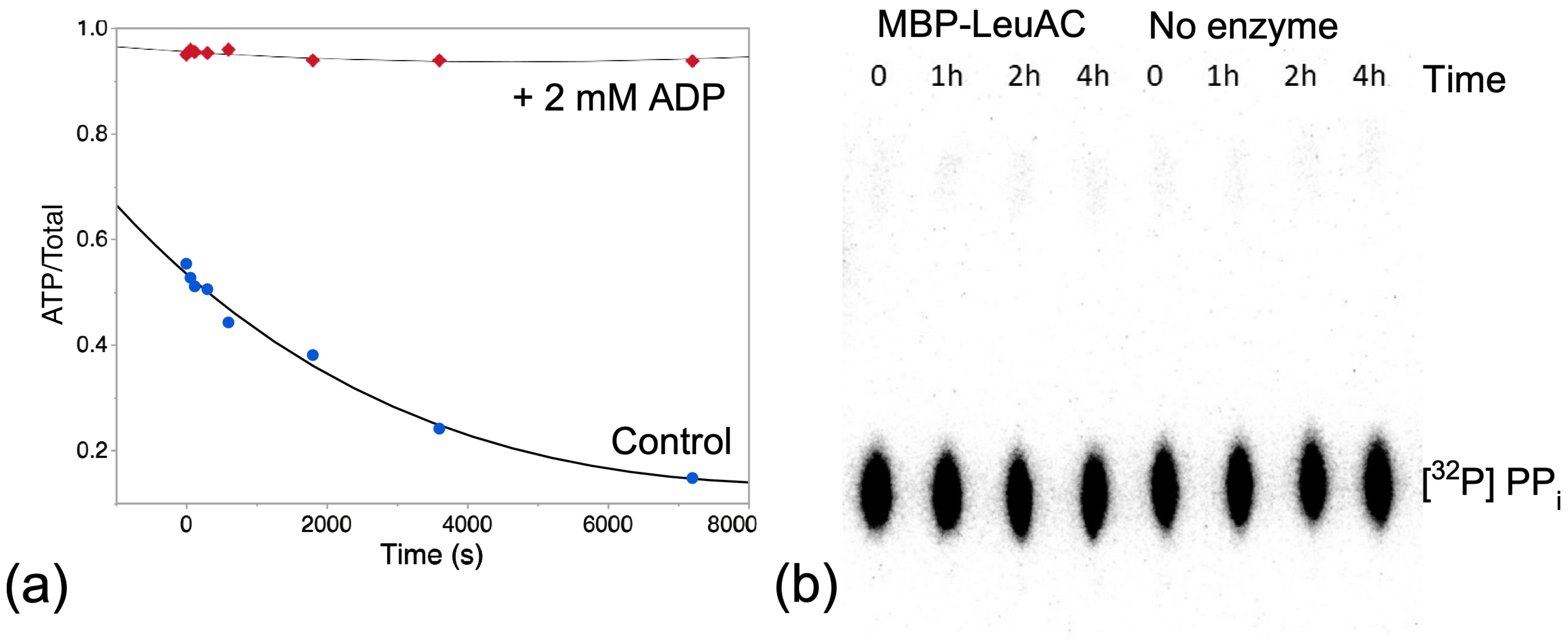

Figure S3. Controls cited in the manuscript that limit interpretations of the unexpected high ADP production in active-site titration assays. **(a)** High (2 mM) ADP concentrations completely inhibit ATP consumption (control). **(b)** LeuAC does not exhibit pyrophosphatase activity. There is no generation of labeled orthorphoshate in the presence of LeuAC.

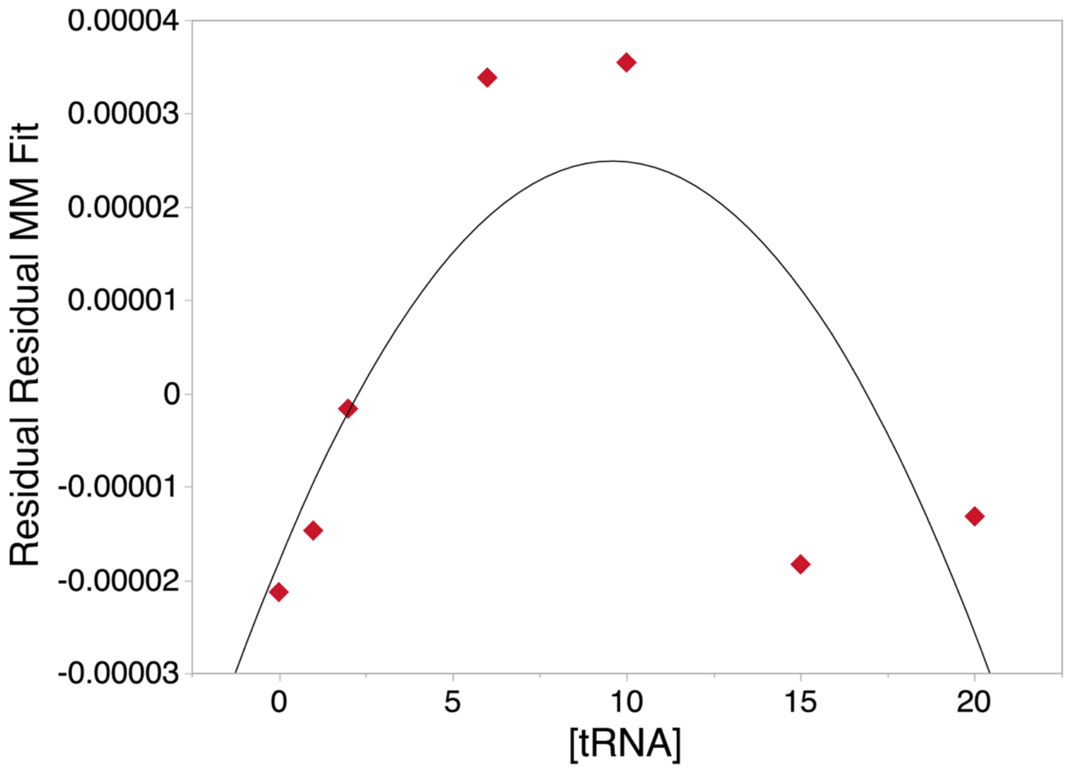

Parameter	β	σ	β/σ
a	-0.0000005	0.0000002	-2.49
b	0.0000089	0.0000037	2.40
c	-0.0000179	0.0000127	-1.41

Figure S4. LeuAC acylation rates at different tRNA^{Leu} concentrations were fitted to both Michaelis-Menten and linear models. The MM model residuals were not correlated with [tRNA] (R^2 = 0.0, 0.008 for linear and quadratic dependences); residuals for the linear model deviated systematically with respect to [tRNA^{Leu}], with smaller values at the two extremes (R^2 = 0.4 for a quadratic dependence). A linear fit between residuals from the two models produced residuals that were fitted to the simple quadratic model shown here, which is statistically significant (R^2 = 0.61 and both a and b coefficients are more than twice the estimated error). Thus, although the Michaelis-Menten fit was only marginally more satisfactory than the linear fit, the pattern of residuals supports the existence of curvature characteristic of saturation behavior in the experimental dependence on [tRNA].

Table S1. Design matrix for analysis of biphasic (single turnover) fits ^{32}P-ATP consumption. Free energies are computed as $\Delta G = -0.592*\ln(k)$ and are in kcal/mole.

**Sample**	**n**	**Date**	**C**	**A**	**k_{chem}**	**k_{cat}**	**k_{chem}/k_{cat}**	**$\Delta G^{\ddagger}k_{chem}$**	**$\Delta G^{\ddagger}k_{cat}$**	**$\Delta Gk_{chem}/k_{cat}$**	**URZ**	**TEV**	**α labeled**	**LSA**	**AMPcPP**	***P. horik***
Ph LeuRS	1.2	6/2/21	0.68	0.32	0.08085	8.97E-05	902	1.49	5.52	-4.03	0	0	0	0	0	1
Ph LeuRS	1.3	5/25/21	0.63	0.37	0.06870	1.59E-04	431	1.59	5.18	-3.59	0	0	0	0	0	1
Ec LeuRS	1.0	12/14/20	0.13	0.29	0.00178	7.06E-06	252	3.75	7.02	-3.27	0	0	0	0	0	0
Ec LeuRS	1.6	12/14/20	0.13	0.44	0.00052	4.30E-06	120	4.48	7.32	-2.83	0	0	1	0	1	0
Ec LeuRS	1.4	12/14/20	0.29	0.39	0.00082	4.42E-06	185	4.21	7.30	-3.09	0	0	1	1	0	0
Ec LeuRS	0.9	12/14/20	0.12	0.24	0.00236	6.05E-06	389	3.58	7.11	-3.53	0	0	1	0	0	0
Ec LeuRS	1.0	12/14/20	0.13	0.29	0.00178	7.06E-06	252	3.75	7.02	-3.27	0	0	1	0	0	0
LeuAC (MBP)	2.4	8/9/18	0.26	0.68	0.00043	2.18E-05	20	4.59	6.35	-1.76	1	0	0	0	0	1
LeuAC (MBP)	2.4	5/25/21	0.26	0.68	0.00043	2.18E-05	20	4.59	6.35	-1.76	1	0	0	0	0	1
LeuAC (MBP)	2.2	12/14/20	0.43	0.62	0.00051	1.19E-05	43	4.49	6.71	-2.22	1	0	1	0	1	1
LeuAC (MBP)	2.5	12/14/20	0.37	0.69	0.00036	4.43E-06	82	4.69	7.30	-2.61	1	0	1	0	0	1
LeuAC (MBP)	2.0	12/4/20	0.42	0.57	0.00033	4.36E-06	75	4.75	7.31	-2.55	1	0	1	0	0	1
LeuAC (MBP)	2.3	12/18/20	0.35	0.65	0.00040	4.27E-06	93	4.64	7.32	-2.68	1	0	1	0	0	1
LeuAC (MBP)	2.5	12/18/20	0.31	0.69	0.00036	*ND*	*ND*	4.69	*ND*	*ND*	1	0	1	0	0	1
LeuAC (MBP)	2.4	12/18/20	0.34	0.67	0.00038	*ND*	*ND*	4.67	*ND*	*ND*	1	0	1	0	0	1
LeuAC (MBP)	2.4	12/18/20	0.33	0.67	0.00037	*ND*	*ND*	4.67	*ND*	*ND*	1	0	1	0	0	1
LeuAC (MBP)	2.3	12/18/20	0.37	0.64	0.00041	*ND*	*ND*	4.62	*ND*	*ND*	1	0	1	0	0	1
LeuAC (MBP)	2.3	12/18/20	0.35	0.65	0.00042	*ND*	*ND*	4.60	*ND*	*ND*	1	0	1	0	0	1
LeuAC (MBP)	2.4	12/18/20	0.34	0.66	0.00037	*ND*	*ND*	4.68	*ND*	*ND*	1	0	1	0	0	1
LeuAC (TEV)	1.9	5/25/21	0.41	0.52	0.00123	2.33E-05	53	3.97	6.31	-2.35	1	1	0	0	0	1
LeuAC (TEV)	2.1	5/4/21	0.27	0.60	0.00326	2.00E-05	161	3.39	6.41	-3.01	1	1	1	0	0	1
LeuAC (TEV)	2.2	5/4/21	0.26	0.62	0.00321	2.07E-05	155	3.40	6.38	-2.99	1	1	1	0	0	1
LeuAC (TEV)	2.2	10/19/17	0.42	0.61	0.00070	1.30E-05	52	4.30	6.66	-2.34	1	1	0	0	0	1
LeuAC (TEV)	2.1	10/19/17	0.43	0.59	0.00066	1.80E-05	37	4.34	6.47	-2.14	1	1	0	0	0	1
LeuAC (TEV)	2.1	5/4/21	0.27	0.58	0.00331	1.97E-05	168	3.38	6.41	-3.03	1	1	0	0	0	1
LeuAC (TEV)	1.9	8/9/18	0.41	0.52	0.00123	2.33E-05	53	3.97	6.31	-2.35	1	1	0	0	0	1

ND denotes kcat values that were determined with $\beta/\sigma < 1.0$

Table S2. Design matrix derived from biphasic (single turnover) fits to aminoacylation experiments Free energies are computed as $\Delta G = -0.592*\ln(k)$ and are in kcal/mole.

**Sample**	**Date**	**C**	**A**	**k_{chem}**	**k_{cat}**	**k_{chem}/k_{cat}**	**$\Delta G^{‡}k_{chem}$**	**$\Delta G^{‡}k_{cat}$**	**$\Delta G k_{chem}/k_{cat}$**	**URZ**	**TEV**	**[tRNA], M**	**[enzyme], M**	**tRNA/E**	**^{14}CLeu**
Ph LeuRS	9/30/20	0.012	0.90	0.0083	1.77E-05	466	2.84	6.48	-3.64	0	0	5.00E-06	5.00E-07	10.0	0
Ph LeuRS	9/24/20	0.001	0.97	0.0026	3.10E-05	83	3.54	6.15	-2.61	0	0	1.00E-05	5.00E-06	2.0	0
Ph LeuRS	9/24/20	0.291	0.68	0.0036	1.53E-04	23	3.34	5.20	-1.87	0	0	1.00E-05	5.00E-06	2.0	0
Ph LeuRS	10/5/20	0.355	0.32	0.0100	3.80E-04	27	2.72	4.66	-1.95	0	0	5.33E-06	5.00E-06	1.1	0
Ph LeuRS	11/23/20	0.084	0.93	0.0046	3.51E-05	130	3.19	6.07	-2.88	0	0	3.24E-05	5.00E-06	6.5	1
Ph LeuRS	11/17/20	0.694	0.31	0.0181	3.19E-04	57	2.38	4.77	-2.39	0	0	1.60E-05	2.60E-05	0.6	1
Ph LeuRS	10/7/20	0.056	0.94	0.0031	3.96E-05	77	3.43	6.00	-2.58	0	0	5.00E-06	1.25E-05	0.4	0
Ph LeuRS	10/7/20	0.137	0.86	0.0041	7.18E-05	57	3.26	5.65	-2.39	0	0	5.00E-06	1.25E-05	0.4	0
Ph LeuRS	10/7/20	0.092	1.15	0.0051	6.68E-05	77	3.13	5.69	-2.57	0	0	4.30E-05	1.25E-05	3.4	0
Ph LeuRS	10/7/20	0.278	0.49	0.0046	1.45E-04	31	3.19	5.23	-2.04	0	0	4.30E-05	1.25E-05	3.4	0
Ph LeuRS	10/7/20	0.356	0.53	0.0041	1.85E-04	22	3.26	5.09	-1.83	0	0	4.30E-05	1.25E-05	3.4	0
Ph LeuRS	10/28/20	0.096	0.90	0.0538	1.97E-04	274	1.73	5.05	-3.32	0	0	2.60E-05	1.30E-05	2.0	1
Ph LeuRS	10/28/20	0.201	0.80	0.0819	2.01E-04	407	1.48	5.04	-3.56	0	0	2.60E-05	1.30E-05	2.0	0
Ph LeuRS	10/9/20	0.292	0.70	0.0440	2.43E-04	179	1.86	4.93	-3.07	0	0	4.60E-08	1.25E-05	0.0	0
Ph LeuRS	10/9/20	0.384	0.60	0.0240	1.84E-04	128	2.22	5.09	-2.87	0	0	1.67E-07	1.25E-05	0.0	0
Ph LeuRS	10/9/20	0.044	0.91	0.0150	7.00E-05	219	2.48	5.67	-3.19	0	0	2.80E-06	1.25E-05	0.2	0
Ph LeuRS	10/6/20	0.016	1.01	0.0092	5.28E-06	1744	2.78	7.19	-4.42	0	0	5.33E-06	5.00E-06	1.1	0
Ph LeuRS	10/6/20	-0.021	0.90	0.0053	6.32E-06	831	3.11	7.09	-3.98	0	0	5.33E-06	5.00E-06	1.1	0
Ph LeuRS	10/6/20	0.180	0.61	0.0063	9.82E-05	64	3.01	5.46	-2.46	0	0	5.33E-06	5.00E-06	1.1	0
Ph LeuRS	10/6/20	-0.153	0.75	0.0053	6.00E-05	88	3.11	5.75	-2.65	0	0	5.33E-06	5.00E-06	1.1	0
LeuAC (MBP)	9/4/19	0.379	0.55	0.0007	9.00E-05	8	4.32	5.52	-1.20	1	0	6.00E-05	5.00E-06	12.0	0
LeuAC (MBP)	3/12/21	0.191	0.81	0.0010	5.48E-05	18	4.11	5.81	-1.70	1	0	9.13E-05	9.10E-06	10.0	0
LeuAC (MBP)	3/24/19	0.230	0.60	0.0029	2.50E-05	116	3.46	6.27	-2.82	1	0	6.00E-05	5.00E-06	12.0	0
LeuAC (MBP)	4/26/21	0.157	0.85	0.0023	5.91E-05	38	3.61	5.76	-2.16	1	0	8.02E-05	3.60E-05	2.2	0
LeuAC (TEV)	3/12/21	-0.017	1.02	0.0021	5.44E-06	387	3.65	7.18	-3.53	1	1	9.13E-05	5.40E-06	16.9	0
LeuAC (TEV)	2/25/19	0.414	0.34	0.0039	3.00E-04	14	3.28	4.84	-1.56	1	1	1.50E-05	5.00E-06	3.0	0
LeuAC (TEV)	3/29/19	0.526	0.47	0.0270	2.48E-04	108	2.15	4.92	-2.77	1	1	5.00E-06	5.00E-06	1.0	0
LeuAC (TEV)	3/22/19	0.258	0.74	0.0491	8.47E-04	58	1.79	4.19	-2.40	1	1	6.00E-05	5.00E-06	12.0	0

Supplementary Table S3. Design matrix for comparing amino acid activation by MBP-fusion and TEV-cleaved wild type and AMSAS mutant LeuAC. Values are given for fitting single-turnover rate measurements for ATP consumption to Eqn (1) of Section 4.5. Free energies are computed as ΔG = –0.592*ln(k) and are in kcal/mole.

Catalyst	C	A	kchem, /s	kcat, /s	kchem/kcat	KMSKS	TEV	ΔG‡kchem	ΔG‡kcat	ΔGkchem/kcat
TEV-AMSAS 26/1/22	0.493	0.348	0.0013	0.000037	34.6	0	1	3.94	6.04	-2.10
MBP_AMSAS_26/1/22	0.385	0.440	0.0014	0.000028	51.3	0	0	3.88	6.22	-2.33
TEV-cleaved_WT 4/5/21	0.274	0.580	0.0033	0.000020	167.9	1	1	3.38	6.41	-3.03
TEV-cleaved_WT 26/10/22	0.279	0.579	0.0034	0.000019	176.3	1	1	3.37	6.44	-3.06
MBP_8/30/21	0.441	0.394	0.0013	0.000029	46.3	1	0	3.93	6.20	-2.27
MBP_WT_15-2-22	0.585	0.309	0.0021	0.000032	67.1	1	0	3.64	6.13	-2.49
MBP_AMSAS_15-2-22	0.525	0.340	0.0022	0.000029	74.9	0	0	3.63	6.18	-2.55
MBP_KMSAS_16-2-22	0.551	0.346	0.0018	0.000029	62.3	1	0	3.75	6.19	-2.45
MBP_AMSAS_16-2-22	0.517	0.349	0.0019	0.000029	67.4	0	0	3.70	6.20	-2.49

Supplementary Table S4. Design matrix for comparing $tRNA^{Leu}$ aminoacylation by TEV-cleaved wild type and AMSAS mutant LeuAC. Aminoacylation rates were determined at [LeuAC] = 1.6 μM and [$tRNA^{Leu}$] = 3 μM. Free energies are computed as $\Delta G = -0.592 * \ln(k)$ and are in kcal/mole.

Rate	$\Delta G^{\ddagger}$(Rate)	KMSKS
0.00031	4.78	1
0.00031	4.78	1
0.00027	4.87	1
0.00027	4.86	1
0.00029	4.81	1
0.00014	5.24	0
0.00022	4.97	0
0.00017	5.12	0

References

1. Pham, Y., Li, L., Kim, A., Erdogan, O., Weinreb, V., Butterfoss, G., Kuhlman, B. and Carter, C.W., Jr. (2007) A Minimal TrpRS Catalytic Domain Supports Sense/Antisense Ancestry of Class I and II Aminoacyl-tRNA Synthetases. *Mol Cell*, **25**, 851-862.
